# Klinischer Verlauf und Diagnostik bei einem Patienten mit Affenpocken

**DOI:** 10.1007/s00105-022-05079-1

**Published:** 2022-11-21

**Authors:** Carolina Laetitia Fiederer, Stephan Forchhammer, Martin Schaller, Simon Riel, Alexander Scheu, Saskia Maria Schnabl

**Affiliations:** Universitäts-Hautklinik Tübingen, Eberhard-Karls-Universität Tübingen, Tübingen, Deutschland Liebermeisterstr. 25, 72076

## Anamnese

In unserer Ambulanz stellte sich notfallmäßig ein 31-jähriger Patient mit seit 5 Tagen bestehendem Fieber, Schüttelfrost und juckendem Exanthem vor. Außerdem waren bereits vor 10 Tagen nach ungeschütztem Sexualkontakt (MSM) schmerzhafte perianale Hautveränderungen aufgefallen. Der Patient berichtete über wechselnde Sexualpartner und eine Vormedikation mit Emtricitabin und Tenofovir als HIV-Präexpositionsprophylaxe. Die Einnahme neuer Medikamente wurde verneint. Eine frühere Luesinfektion wurde laut Patient ausreichend therapiert. Ein Auslandsaufenthalt war nicht vorangegangen.

## Untersuchung

**Figure 1:**
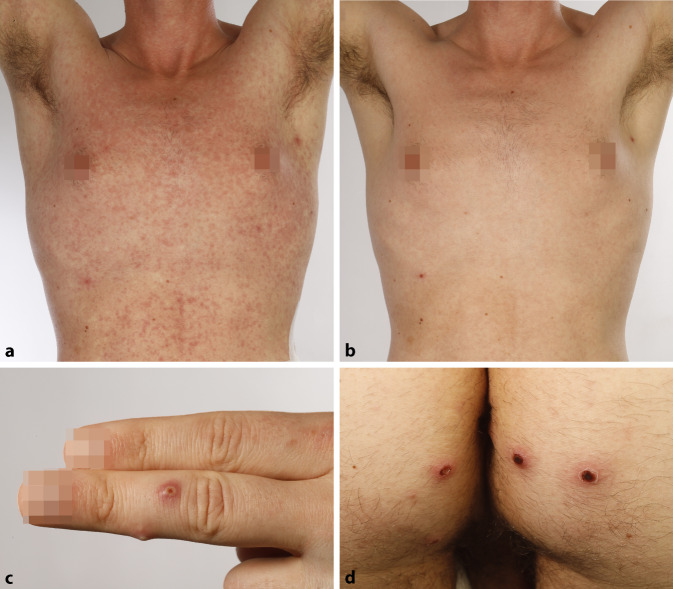

Klinisch bestanden bei Erstvorstellung ein stammbetontes makulopapulöses Exanthem, teilweise mit konfluierenden Effloreszenzen, und mehrere perianale Knoten mit Erosionen, Krusten und Nekrose (Abb. 1a, d).

## Diagnostik

**Figure 2:**
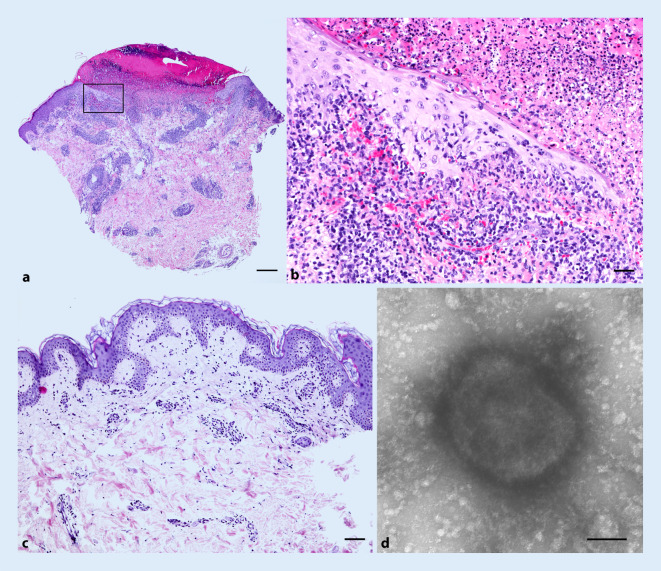

Laborchemisch zeigten sich geringgradig erhöhte Entzündungsparameter, eine HIV-Serologie verblieb negativ. In der Luesserologie zeigte sich eine Seronarbe ohne Anhalt für eine frische Infektion bei Zustand nach therapierter Luesinfektion in der Vergangenheit. Eine PCR-Untersuchung ergab keinen Nachweis einer Herpes-simplex-Infektion (HSV 1 und 2). Das histologische Ergebnis einer Stanzbiopsie vom Abdomen zeigte eine superfiziell perivaskuläre lymphozytäre Dermatitis, vereinbar mit einem infekt- oder arzneimittelgetriggerten Exanthem (Abb. 2c).

## Therapie und Verlauf

Unter einer aufgrund der initial bestehenden Verdachtsdiagnose eines infektgetriggerten Erythema exsudativum multiforme bei perianaler Herpes-simplex-Infektion eingeleiteten systemischen Therapie mit Prednisolon (0,5 mg/kgKG) über 3 Tage kam es zu einer raschen Abheilung des Exanthems. Am Tag des Absetzens traten allerdings am gesamten Integument einzeln stehende Pusteln mit erythematösem Randsaum (Abb. 1b, c), eine schmerzhafte zervikale Lymphknotenschwellung, Schluckbeschwerden und enorale Erosionen auf. Eine PCR-Untersuchung des Pustelinhalts konnte die Verdachtsdiagnose einer Infektion mit Affenpocken bestätigen. Eine erneute Stanzbiopsie einer Pustel am linken Ellenbogen zeigte eine zentrale Ulzeration mit hämorrhagischer Kruste. Im Randbereich des Ulkus fanden sich in der Epidermis vereinzelte Viruseinschlusskörper sowie eine Interface-Reaktion mit dichten subepidermalen lymphohistiozytären Infiltraten (Abb. 2a, b). Elektronenmikroskopisch konnten quaderförmige Orthopox-Viren mit maulbeerartiger Oberflächenkonfiguration, passend zu Affenpocken, dargestellt werden (Abb. 2d). Eine Meldung an das zuständige Gesundheitsamt erfolgte bereits bei Verdacht. Der Patient wurde isoliert und musste sich nach Entlassung insgesamt für 21 Tage in häusliche Quarantäne begeben. Perianal bestanden weiterhin stark nässende Erosionen. Eine im Verlauf durchgeführte proktoskopische Untersuchung zeigte eine ausgeprägte erosive Proktitis mit Fibrinbelägen. Eine symptomatische Therapie mit Jelliproct und Xylocain-Gel wurde eingeleitet.

## Diskussion

Affenpocken sind bereits seit 1958 bekannt und wurden damals aus infizierten Affen isoliert [3]. Die erste Infektion eines Menschen wurde 1970 im Kongo bestätigt [5]. Die Ausbreitung des Erregers ist seitdem in Zentral- und Westafrika endemisch [3]. Immer wieder kam es auch in der westlichen Hemisphäre zu Fällen, welche jedoch regelhaft in Zusammenhang mit dem Handel exotischer Tiere oder internationalen Reisen standen. Verursacht wird die Erkrankung durch ein Orthopox-Virus. Überträger sind tierische Wirte (Nager, u. a. Ratten, Eichhörnchen, Mäuse) [10]. Es gibt genetisch unterschiedliche westafrikanische und zentralafrikanische Varianten, die auch eine unterschiedliche Virulenz aufweisen. Entsprechende Varianten können durch PCR-Untersuchungen und Sequenzierungen in Speziallaboratorien unterschieden werden [2, 10, 11].

Bei dem aktuellen Ausbruch der Affenpocken wurden bereits in mehr als 30 Ländern in Europa, Nord- und Südamerika, Australien und dem Nahen Osten Infektionen bestätigt. Bis zum 07.06.2022 konnten 1066 Fälle bestätigt werden [1]. Die Häufung der Infektionen begann im Mai 2022 und betrifft wie auch in dem von uns beschriebenen Fall v. a. junge Männer, die ungeschützten Sex mit Männern haben, dementsprechende Gruppen oder Großveranstaltungen besuchen und vorher nicht in endemische Gebiete in Afrika gereist waren [7, 9]. Untersuchungen konnten genetisch Homologien zur gutartigeren westafrikanischen Variante nachweisen [11]. Das Alter der betroffenen Patienten ist eher jung, auch weil ältere Menschen (Jahrgang 1976 in westlichen Bundesländern und 1982 in östlichen Bundesländern und älter) aufgrund der damals bestehenden Impfpflicht einen relativen Impfschutz durch Pockenimpfungen haben [1].

Die klinischen Verläufe sind hier sehr unterschiedlich und können von monosymptomatisch bis disseminiert oder selten mit Komplikationen verlaufen [9]. Die häufigsten klinischen Symptome sind Fieber (54 %), Exantheme (40 %), Lymphknotenschwellungen (46 %), Kopfschmerzen (26 %), Müdigkeit (23 %) und Myalgien (17 %) [1, 6]. Neu beschriebene klinische Erscheinungsformen sind Penisödeme und rektale Schmerzen [9]. Außerdem treten genitale und anale Läsionen (Erosionen und Bläschen) und im Verlauf Pusteln auf [1, 6]. Die Pusteln ähneln morphologisch den Pocken, haben häufig einen erythematösen Randsaum und können erosiv oder nekrotisch werden. Sie lösen Juckreiz, aber auch Schmerzen aus und heilen unter Narbenbildung ab [6, 8]. Die ersten Effloreszenzen treten an der Lokalisation der Exposition auf. Der Übertragungsweg erfolgt bei engem Körperkontakt, am ehesten in Form einer Schmier- oder Tröpfcheninfektion. Im Verlauf kommt es dann meist zur Lymphknotenschwellung und Generalisierung mit Auftreten eines Exanthems und weiteren spezifischen Hautläsionen. Die Inkubationszeit beträgt 5 bis 21 Tage. Der Verlauf ist häufig selbstlimitierend. Die Infektiosität stimmt mit dem Beginn der Symptome überein, und daher müssen enge Kontakte nicht isoliert werden, während sie asymptomatisch sind [8]. Patienten gelten nicht mehr als infektiös, nachdem alle Krusten abgefallen sind [12]. Die Diagnosestellung erfolgt klinisch und wird durch histologische und labordiagnostische Untersuchungen, z. B. Elektronenmikroskopie, ergänzt. Laut WHO wird eine Bestätigung der Diagnose mittels PCR aus den Läsionen empfohlen [8].

Die Behandlung ist symptomorientiert. Bei stark symptomatischen Verläufen gibt es Therapieversuche mit Tecovirimat und Brincidofovir [6]. Schwere Komplikationen sind bei der aktuellen Variante selten und treten v. a. bei Pocken-ungeimpften Patienten auf [7]. Seltene beschriebene Komplikationen sind Bronchopneumonien, Erbrechen und Durchfall mit schwerer Dehydrierung, Enzephalitis und Sepsis [4].

## Fazit für die Praxis

Dieser aktuelle Fall veranschaulicht sehr gut den klinischen Verlauf der einzelnen Stadien und die histologischen und elektronenmikroskopischen Ergebnisse bei dem in Deutschland neu aufgetretenen dermatologischen Krankheitsbild der Affenpockeninfektion. Aufgrund der aktuell stark ansteigenden Fallzahlen sollte bei passender Anamnese bei unspezifischem Fieber oder einem Exanthem an eine Infektion mit Affenpocken gedacht werden und v. a. bei monomorphem Wechsel der Hauteffloreszenzen eine erneute Diagnostik erfolgen. Entsprechenden Risikogruppen sollte eine Impfung angeboten werden.
